# Evaluation of the Individual, Relationship, and Financial Benefits of Juntos en Pareja for Spanish‐Speaking Latine Couples

**DOI:** 10.1111/famp.70153

**Published:** 2026-05-14

**Authors:** Mariana K. Falconier, Hannah C. Williamson, Zeinab Azizi, Martha Yumiseva‐L

**Affiliations:** ^1^ Department of Family Science University of Maryland College Park Maryland USA; ^2^ Department of Human Development and Family Sciences University of Texas at Austin Austin Texas USA

**Keywords:** financial education, intimate relationships, Latine couples, relationship education

## Abstract

Latine couples often face high levels of financial stress, which can significantly strain romantic relationships. Yet few programs are designed to address both financial and relational distress for this population. Juntos en Pareja (*JEP*) is a socio‐culturally responsive program designed to address these challenges by providing relationship and financial education to Spanish‐speaking Latine couples. *JEP* is a cultural and linguistic adaptation of the evidence‐based program TOGETHER. This study evaluates *JEP'*s effectiveness by comparing pre‐ and post‐workshop outcomes among Latine couples in *JEP* with a comparison group of non‐Latine couples in the TOGETHER program. We examined three domains: psychological well‐being, relationship quality, and financial stability. The analytic sample included 284 couples (568 individuals) in *JEP* and 266 couples (532 individuals) in TOGETHER, all residing in the United States. Using linear mixed models, we assessed changes from pre‐ to post‐test across key indicators. Similar to TOGETHER, *JEP* participants showed significant declines in psychological distress, negative conflict management, and psychological aggression toward the partner and by the partner, and significant increases in time spent with the partner. Additionally, *JEP* couples also reported reduced difficulty in paying bills. These results provide support for *JEP* as a beneficial program for Spanish‐speaking Latine couples, but they should be considered with caution as they represent the experience of program completers with high attendance and immediately upon program completion. Further studies should assess the sustainability of changes over time. Nonetheless, these initial findings underscore the need for accessible, socio‐culturally attuned interventions to promote resilience and stability in underserved immigrant communities.

## 1 Introduction

Financial concerns have been a dominant stressor for U.S. adults for decades, with approximately 67% reporting significant stress related to covering essential expenses in recent years (American Psychological Association 2023). This financial strain disproportionately affects the Latine population due to persistent income and wealth disparities (Lin et al. 2025). In 2023, the median household income for Latine families was $69,467, the second lowest after Black households at $53,493, and significantly lower than the median household incomes for non‐Latine white households ($83,121) and Asian households ($111,366; U.S. Census Bureau 2023). Within the Latine population, the household income for the foreign‐born is more than 10% lower than for U.S.‐born (Moslimani and Noe‐Bustamante 2023). Latines, particularly first‐generation immigrants, tend to have limited savings, minimal emergency funds, fewer financial assets, restricted access to financial assistance, and lower levels of financial literacy (Vargas and Sanchez 2020). Latine households may struggle with housing payments and resort to high‐risk strategies, such as delaying education and healthcare or relying on payday loans (Vargas and Sanchez 2020). These financial challenges are often compounded by intersecting systemic inequities, including racism, nativism, and xenophobia, that limit access to employment, education, healthcare, and other essential services (Salinas and Salinas 2022; Samari et al. 2024).

Financial stress exerts a substantial toll on physical health, evidenced by its association with an elevated risk of chronic pain (Weissman et al. 2023) and major cardiac events (Swarup et al. 2024) and dietary risk (Du et al. 2021), among other adverse outcomes. Studies, including those on Latine individuals specifically, indicate that the detrimental impact of financial hardship also extends to mental well‐being, as it has been linked to depressive symptoms (Falconier 2010), anxiety (Villatoro et al. 2022), alcohol and substance use (Cerezo et al. 2024), and suicidal ideation and attempts (Elbogen et al. 2020).

In addition to its negative impact on individual well‐being, economic stress also negatively affects couple relationships. A recent meta‐analysis, including studies on Latine populations (Falconier and Jackson 2020), indicated that financial stress is associated with increased negative interactions, such as emotional abuse and conflict, decreased positive interactions, such as gratitude and constructive conflict resolution, and elevated relationship instability and dissatisfaction. These associations were found to be more pronounced among low‐income dyads, suggesting that objective economic difficulties may amplify the detrimental effects of financial stress on relationships. In the case of the Latine population, especially first generation immigrants, the impact of the financial stressors on individual and relational well‐being is exacerbated by other everyday chronic stressors such as discrimination, language barriers, cultural differences, immigration status, and limited access to health care, education, and job opportunities, among others (Falconier et al. 2013). This is why understanding the structural inequities that Latine low‐income couples experience in the U.S. is critical to fully understand their challenges to overcome their financial difficulties and the interaction of the multiple stressors that they experience in their daily lives.

While financial difficulties impact couple functioning, relationship quality also predicts sound financial management and effective financial communication (Saxey et al. 2023). This interplay between relationship functioning and financial management has led to the development of couple financial therapy, a therapeutic approach addressing both relational and financial challenges (Ford et al. 2020). Similarly, psycho‐educational initiatives like the TOGETHER program, developed during the 2008 recession, aim to support couples in managing their relational and financial concerns (Falconier 2015).

TOGETHER is an evidence‐based program integrating relationship and financial education to enhance individual, relational, and financial well‐being in couples (Falconier, Kim, et al. 2023). Unlike other couple relationship education (CRE) programs such as ELEVATE (Futris et al. 2014), PREP (Markman et al. 2010), OurRelationship (Doss et al. 2013), and Hold Me Tight (Johnson 2009), TOGETHER uniquely emphasizes stress management skills at both individual and dyadic levels as well as financial management. Existing CRE programs for couples often inadequately address financial stressors and fail to integrate financial literacy as a core component. When financial topics are included, they are typically relegated to a single module within a broader curriculum (Scott and Huz 2020). In fact, many CRE programs have faced criticism for their limited effectiveness with couples from low‐income and racial or ethnic minority backgrounds (Bradbury and Lavner 2012; Arnold and Beelmann 2019). TOGETHER addresses this gap by offering a group‐based approach that focuses on financial stress, providing an affordable and accessible alternative to couple financial therapy for economically disadvantaged couples.

Available curricula for first‐generation immigrant Latine couples are often translations of existing CRE programs, such as Within our Reach (Rhoades 2015) and Gottman's Bringing Baby Home curriculum (Shapiro et al. 2011). Even though translations of CRE curricula remove the language barrier for first‐generation immigrant Latine couples for whom language is an important barrier to accessing this service, they may not be socio‐culturally attuned to this population. The curricula may ignore the cultural values, the lived experiences, and the contextual reality of the Latine community in the U.S. Such an approach may miss the opportunity to empower these couples and value their strengths (e.g., collective ways of coping) and resilience (e.g., collective ways of coping, expression of emotions, family loyalty and commitment, etc.).

Recognizing this gap and to provide a more socio‐culturally attuned program to Latine couples, the TOGETHER curriculum was linguistically and culturally adapted into the Juntos en Pareja (*JEP*) program. *JEP* includes material that is congruent with Latines' cultural values and addresses aspects specific to their immigration experience in the areas of stress and financial management (see later section on *JEP* for a description of curriculum modifications). Most importantly, *JEP* seeks to empower Spanish‐speaking Latine couples by presenting and discussing the material in a way that is understanding of their circumstances, respectful of the values and traditions that they embrace, and honors their wisdom and identities, and by providing critical information to navigate the financial system in the U.S. and improve their chances to strengthen their financial situation.

*JEP* was initially pilot‐tested with 23 Latine immigrant couples, and preliminary outcomes suggested potential program effectiveness in reducing financial stress and emotional dysregulation while improving financial management, relationship satisfaction, and dyadic coping (Yumiseva et al. 2025). The present study aimed to evaluate *JEP*'s short‐term effectiveness in improving Spanish‐speaking Latine couples' psychological well‐being, relationship functioning, and financial management with a larger sample. The evaluation compared changes in pre‐ and post‐program participation between *JEP* couples and non‐Latine couples in the TOGETHER program. The post‐program assessment took place immediately upon program completion, and funding restrictions prevented any further follow‐up assessment to evaluate the sustainability of program outcomes.

While this study focuses on shared experiences among Spanish‐speaking Latine couples, it is important to acknowledge that Latine communities are not a monolith. Differences in country of origin, migration histories, language, and cultural values shape distinct needs and perspectives. Recognizing this heterogeneity is essential to honoring participants' diverse experiences and avoiding overgeneralization in both research and program adaptation.

## 2 The TOGETHER Program

The TOGETHER program is an adaptation to financial stress of the Couples' Coping Enhancement Training (CCET; Bodenmann and Shantinath 2004), a psychoeducational CRE intervention demonstrated to enhance relationship quality, improve couple communication, and strengthen stress management skills (Bodenmann et al. 2008; Ledermann et al. 2007). Similar to CCET, rooted in the systemic transactional model of dyadic coping (Bodenmann 1997) and cognitive behavioral couple therapy (Epstein and Baucom 2002), the TOGETHER program emphasizes effective communication, stress management at individual and dyadic levels, and problem‐solving strategies. Additionally, the program incorporates key concepts from the Theory of Planned Behavior (TPB; Ajzen 1991; Xiao 2008) and social cognitive theory (Bandura 1997) to strengthen couples' financial management skills.

The TOGETHER program is designed to help reduce partners' overall stress and finance‐related stress, while improving their relationship functioning and financial management. To accomplish these objectives, the program provides couples with knowledge and tools to strengthen individual and dyadic stress management, communication and de‐escalation strategies, dyadic problem‐solving skills, and financial management. The TOGETHER curriculum includes nine modules, each blending conceptual and interactive skill‐building content with hands‐on activities such as role‐plays and games, along with take‐home practices (Falconier, Kim, and Lachowicz 2023). The modules include: (a) understanding how general and financial stress affect individuals and relationships, (b) strategies for managing personal and financial stress, (c) communicating stress and financial concerns with a partner, (d) dyadic stress and financial stress management, (e) discussing finances and other sensitive topics, (f) examining relationship with money and financial roles within the relationship, (g) foundational financial management, (h) credit management, and (i) financial problem‐solving (see names of modules in Table S1 and for a detailed description of each module, refer to Falconier 2015). The workshops are held in small groups of 4 to 10 couples and take place over weekly sessions, co‐facilitated by a couple expert (e.g., couple therapist) and a financial expert (e.g., financial counselor). The workshops could be delivered in two formats: either four weekly sessions of 4.5 h each (total: 18 h over 4 weeks) or eight sessions of 2.5 h each (total: 20 h over 8 weeks). The slight difference in total hours accounts for the review, warm‐up, and break times included in every session (for a more detailed description, see Falconier 2015).

### 2.1 Effectiveness of the TOGETHER Program

Following the initial pilot test (Falconier 2015), the TOGETHER program's effectiveness was evaluated in various studies. A randomized controlled trial (RCT) showed that upon program completion participants showed significant improvements compared to the no‐intervention control group in individual coping strategies with financial stressors and in general, positive and negative conflict management, relationship satisfaction and commitment, common dyadic coping, financial dyadic coping, and general stress (Falconier, Kim, and Lachowicz 2023) and these changes were maintained at the 6‐month follow‐up. A subsequent study evaluating a shorter version of the program (14 h) showed that it was as effective as the longer 8‐week 20‐h workshop and significantly better than a no‐intervention control group in strengthening relationship quality, satisfaction, commitment, and positive conflict management and in reducing negative conflict management and difficulty paying bills. The 14‐h version also had lower dropout rates and higher participation and attracted more participants with low incomes (Falconier, Foss, et al. 2023).

More recently, the online version of the 14‐h curriculum was evaluated by comparing pre‐ and post‐intervention changes with the in‐person program (Azizi et al. 2025). Results indicated that the online program was as effective as the in‐person program in reducing psychological distress and negative conflict management while improving positive conflict management, conflict management satisfaction, relationship quality and satisfaction, and shared time with the partner. Unlike the in‐person workshops, online participants did not report a decrease in difficulty paying bills, even though their levels of satisfaction with the financial component of the program were high. The online and in‐person workshops had comparable retention rates.

### 2.2 The Juntos en Pareja (JEP) Program

The *JEP* program has retained TOGETHER's nine‐module curriculum while incorporating additional content and examples that are socio‐culturally attuned to Latine immigrant couples. To ensure socio‐cultural relevance and appropriateness, modifications of concepts, examples, and activities were guided by Latine cultural specific values (e.g., familismo,^1^ spiritual orientation, gender role differences, personalismo,^2^ collectivism) and understanding of the lived experiences of Latine immigrant couples in the U.S. and contextual factors. These adaptations were made by one of the curriculum developers, an immigrant Latina with over 25 years of therapy and research experience with low‐income Latine immigrant couples, and a Latina immigrant financial counselor with expertise in financial education for this community. The modifications were also informed by relevant literature, including the National Research Center on Hispanic Children and Families guidelines (Wildsmith and Chen 2023). Content modifications to the curriculum included the incorporation of immigration‐related stressors (e.g., acculturation, underemployment, language barriers, discrimination, and separation from family and country of origin) as critical to the experience of stress and specific cultural coping strategies such as collective coping and spiritually oriented strategies.

Modifications to the financial management components were made to: (a) reflect multigenerational household dynamics, including financial contributions from extended family members; (b) address the role of remittances in household finances; and (c) emphasize financial planning and understanding the U.S. credit system, which may differ significantly from financial practices in the couples' home countries. The resulting modified curriculum was translated by a native Spanish‐speaking therapist and a Latina immigrant financial counselor. The translation was further reviewed by the Latina curriculum developer for cultural and linguistic accuracy. Further minor modifications were introduced after the pilot test of *JEP* based on participants' qualitative feedback. The *JEP* curriculum requires facilitators who identify as Latine and understand both the cultural values and immigration reality of the community to manage the presentation of content, examples, and group discussions appropriately and honor participants' values, wisdom, and resilience.

The pilot test of *JEP* (Yumiseva et al. 2025) with 23 Latine immigrant couples indicated a reduction in financial stress and improvement in financial management skills for both partners after program completion and at 3‐month follow‐up. Male participants also experienced a significant decline in emotional regulation difficulties and increased relationship satisfaction. Self‐reported and observational data from video‐recorded couple discussions on individual and common stressors showed improvements in couples' conjoint coping for both partners. Participant satisfaction with the session content, activities, and homework was high, with couple and financial facilitators rated 9.9 and 9.8, respectively, on a 1–10 scale.

### 2.3 The Present Study

Building upon the promising results of the *JEP* pilot test, the current study aimed to evaluate the effectiveness of *JEP* by comparing pre‐ and post‐workshop outcomes among Spanish‐speaking Latine couples participating in *JEP* with those of non‐Latine couples participating in the TOGETHER program. Post‐workshop outcomes were assessed immediately upon workshop completion, as funding restrictions prevented a follow‐up assessment. The evaluation focused on three key areas targeted by the programs: individual psychological well‐being, relationship functioning, and financial management. Regarding psychological well‐being, we hypothesized a reduction in psychological distress following program participation. In terms of relationship functioning, we expected decreases in negative conflict management and psychological aggression (both toward and by partner), and increases in positive conflict management, conflict management satisfaction, relationship quality, and shared time with partner. Concerning financial management, we expected reduced difficulty paying bills. We hypothesized that these changes in the *JEP* group would be comparable to those observed in the TOGETHER group. Additionally, we anticipated similar retention and attendance rates and program satisfaction levels across the *JEP* and TOGETHER programs.

## 3 Method

### 3.1 Participants

Data examined in the current study were drawn from couples participating in *JEP*, who were all Spanish‐speaking Latine, and a comparison group of English‐speaking non‐Latine couples participating in TOGETHER between May 2021 and October 2024. The analytic sample consists of 550 couples (1100 individuals); 284 *JEP* couples (568 individuals) and 266 TOGETHER couples (532 individuals). In the *JEP* group, the primary language spoken in the household was only Spanish for 85% of participants and English and Spanish for 15% of participants, whereas no TOGETHER participant reported Spanish as a primary language spoken in the household. Even though participants' birthplace was not collected, considering that Spanish was a primary language spoken by almost all participants in the household, it is highly likely that the majority of *JEP* participants were first generation immigrants.

*JEP* and TOGETHER program implementation was funded by a Healthy Marriage and Responsible Fatherhood (HMRF) grant (2020–2025) awarded by the Administration of Children and Families, U.S. Department of Health and Human Services. Participants were recruited from the Washington D.C. metropolitan area using multiple outreach strategies. JEP participants were primarily recruited through word‐of‐mouth (35.6%) via Latina health promoters, consistent with Latines' *personalismo* (Evans et al. 2007). Other significant recruitment sources included community organizations (18.1%) and community events (16.8%), with social media accounting for a smaller proportion (7.1%). TOGETHER couples were primarily recruited through word of mouth (33.7%), but had a higher proportion recruited via social media (23%).

Eligibility criteria for both programs required participants to be at least 18 years of age, involved in a committed romantic relationship, and willing to participate in the program with their partner. Exclusion criteria included the presence of severe psychiatric disorders requiring hospitalization within the preceding 12 months or an untreated substance use disorder. Couples were also screened for domestic violence; those in which at least one partner expressed safety concerns were referred to domestic violence services and deemed ineligible for participation. After signing the consent form to participate in the program, no couples had to be excluded due to domestic violence. This may be attributed to the fact that this exclusion criterion was part of the program description that couples had access to through the program's website and other materials prior to providing consent.

### 3.2 Procedure

Both the TOGETHER group and the *JEP* group followed the same procedure. After providing informed consent and screening for domestic violence, couples were enrolled in the program and completed the pre‐treatment survey (T1), just before the beginning of the first workshop session. The post‐treatment survey (T2) was completed at the end of the sixth workshop session. Both assessments were completed by partners separately and by accessing an nFORM‐generated link and passcode for each of them. All enrolled participants were asked to complete the T2 assessment regardless of the number of workshop sessions they had attended and whether they had attended the last session. The assessments included the Information, Family Outcomes, Reporting, and Management (nFORM) survey, mandated by the federal funding agency. Participants in both groups accessed the nFORM website to complete the surveys during an online meeting with program staff. Each participant received $80 in gift cards for survey completion.

Couples in both groups participated in the 14‐h workshop through six synchronous virtual sessions conducted via the Zoom platform (one session per week). The initial session was 1.5 h and followed a one‐hour intake and enrollment meeting, during which participants also completed assessments. The subsequent five sessions were each 2.5 h in duration. Among participants enrolled in TOGETHER, the majority attended 5–6 sessions (70.2%), followed by 1–2 sessions (21.8%). A smaller proportion attended 3–4 sessions (7.6%), and only 0.4% did not attend any sessions. Similarly, among participants in the JEP workshops, most attended 5–6 sessions (59.6%), followed by 1–2 sessions (27.4%), with fewer attending 3–4 sessions (7.3%) or none at all (5.6%). Eighty‐eight TOGETHER workshop series/cohorts and 56 *JEP* workshop series/cohorts were included in the present study.

To ensure fidelity to the curriculum protocol, facilitators completed surveys after each session, were regularly observed by supervisors, and participated in a bi‐weekly supervision meeting. Couples who missed a session received all relevant materials and a video recording of the session.

Every cohort in both groups, TOGETHER and JEP, was assigned a case manager who followed the same procedures. After the first workshop session, the case manager emailed each couple a Qualtrics link to complete a needs assessment survey. The assessment asked couples about their needs across multiple domains, including education, housing, food security, childcare, employment, legal issues, and physical and mental healthcare. The case manager reviewed the couple's responses and emailed them information about affordable or free community services for any identified needs. The case manager subsequently contacted the couple by email or phone to check whether they had followed up on the referral and/or address any barriers that had prevented them from doing so. In addition to providing referrals to resources, case managers also supported program attendance by contacting all participants weekly by email and/or phone to remind them of the upcoming workshop session, to distribute electronic gift cards, and/or to provide further community referrals if needed. Case managers also contacted partners if they missed a workshop session and assisted in resolving any barriers that prevented them from participating. Four hundred and thirty‐six TOGETHER couples (76.76%) and 360 *JEP* couples (67.66%) received at least one referral to community services. The mean for the total number of referrals per participant was 3.6 (SD = 3.28) for the TOGETHER group and 3.51 (SD = 4.86) for the *JEP* group.

The program offered to both groups reimbursement for childcare provided by licensed professionals/organizations. No participants requested such reimbursement. All study procedures were approved by the Institutional Review Boards of the affiliated universities.

### 3.3 Measures

#### 3.3.1 nFORM Survey

The Information, Family Outcomes, Reporting and Management System (nFORM) instrument was developed by Mathematica as part of the Fatherhood and Marriage Local Evaluation Cross‐Site Project, overseen by the Office of Planning, Research, and Evaluation within the Administration for Children and Families, U.S. Department of Health & Human Services (2020). The present study included subscales from the personal development and healthy relationships sections to examine psychological distress, relationship functioning, and financial management.

##### 3.3.1.1 Psychological Distress

Participants were asked to report the extent to which they felt six different symptoms of psychological distress over the past 30 days (e.g., nervous, hopeless, restless or fidgety). The items correspond to the Kessler Psychological Distress Scale (Kessler et al. 2003). Items were scored on a 5‐point scale, with 1 = none of the time and 5 = all of the time. The six items were averaged to form the scale score. Cronbach's alpha was ≥ 0.81 for both groups at both time points.

##### 3.3.1.2 Conflict Management

Positive and negative conflict management were assessed using seven and five items, respectively, measuring adaptive and maladaptive conflict strategies. Adaptive strategies included respectful discussions of disagreements, while maladaptive strategies involved conflicts. Participants reported the frequency of these behaviors over the past month using a 4‐point scale (1 = never, 4 = often). Scale scores were computed by averaging the respective items. For positive conflict management, Cronbach's alpha was ≥ 0.80 across both groups and time points, while for negative conflict management, Cronbach's alpha was ≥ 0.88 across both groups and time points.

##### 3.3.1.3 Psychological Aggression Toward and by Partner

Participants reported how frequently, over the past month, they had blamed their partner for their problems and yelled or screamed at them, as well as how often their partner had done the same to them. These two items were each rated on a 4‐point scale (1 = never, 4 = often). Scores were averaged to create scale scores for both dimensions. Cronbach's alpha was ≥ 0.66 for psychological aggression toward the partner and ≥ 0.78 for psychological aggression by the partner across both groups and time points.

#### 3.3.2 Time Spent With Partner

Participants were asked to report how often in the past month they had engaged in various activities (e.g., “laugh together”) with their partner. Items were scored on a 4‐point scale, with 1 = almost every day, 2 = once or twice a week, 3 = once or twice a month, and 4 = less often. Scores were reverse‐coded such that higher scores indicate more time spent together, and the three items were averaged to form the scale score. Cronbach's alpha was ≥ 0.75 for both groups at both time points.

##### 3.3.2.1 Relationship Quality

This construct was assessed with five items that tapped into global satisfaction with their relationship, such as feeling appreciated and understood by their partner and trusting their partner. Items were scored on a 4‐point scale, with 1 = strongly disagree and 4 = strongly agree. The five items were averaged to form the scale score. Cronbach's alpha was ≥ 0.88 for both groups at both time points.

##### 3.3.2.2 Difficulty Paying Bills

Participants were asked, “How often do you find it difficult to pay your bills?” with response options ranging from 1 = never to 4 = very often.

##### 3.3.2.3 Program Perceptions

At post‐test, participants' perception of the helpfulness of the program was assessed with two items, which asked about the helpfulness of the program overall, as well as helpfulness specifically for financial well‐being. Each item was scored on a five‐point scale, with 1 = not at all and 5 = extremely helpful.

#### 3.3.3 Session Evaluation Questionnaires

Participants' perceptions of the program were also assessed through the Session Evaluation Questionnaire (SEQ), which was collected at the end of the program. This instrument assessed participants' perceptions of the clarity, organization, relevance, appropriateness, and utility of the sessions' content and activities on a four‐point Likert‐type scale, ranging from 1 = strongly agree to 5 = strongly disagree. Additionally, participants assessed facilitators' overall performance using a 10‐point Likert‐type scale, with responses ranging from 1 = poor performance to 10 = excellent performance.

### 3.4 Analytic Plan

To examine change in the outcome variable from pre‐test to post‐test, we used linear mixed models, with one model estimated per outcome. The mixed models account for interdependency in the dyadic data by nesting individuals within couples at Level 1. Treatment group (TOGETHER vs. *JEP*) was used as a between‐couple moderator at Level 2. Following an intent‐to‐treat approach, all available data were included, regardless of dosage or completion of post‐test, which allows for the most accurate estimate of pre‐treatment functioning. The amount of missing data in T1 and T2 measures ranged from 2% to 10%. Missing data were accounted for by averaging across items to create scale scores. The mixed model took the form:

Level 1:

$$Y_{\mathrm{ijt}}=\beta_{0\mathrm{ij}}+\beta_{1\mathrm{ij}}{\text{time}}_{t}+e_{\mathrm{ijt}}.$$

Level 2:

$$\beta_{0\mathrm{ij}}=\gamma_{00}+\gamma_{01}{\text{group}}_{i}+u_{j}+v_{\text{j0}}.$$

$$\beta_{1\mathrm{ij}}=\gamma_{10}+\gamma_{11}{\text{group}}_{i}+v_{\text{j1}}.$$

where *Y*_*ijt*_, the outcome for participant *i* in couple *j* at time *t*. β_0*ij*_, the individual intercept. β_1*ij*_, the individual slope. *γ*_00_, the overall intercept. *γ*_01_, the effect of group on the intercept. *u*_*j*_, the random intercept for couples. *v*_*j0*_, the random intercept for individuals. *γ*_10_, the fixed effect of time (slope). *γ*_11_, the interaction of group X time.

Between‐group differences in treatment effect were examined by decomposing each mixed model into simple slopes for the two treatment groups and testing for between‐group differences in the rate of change over time. Additionally, pre‐test levels of each outcome variable were tested for between‐group differences to examine whether the two groups were starting at similar levels of relational and financial functioning when they entered the intervention. To examine between‐group differences in participants' SEQ evaluations of the program, independent samples *t*‐tests were conducted comparing participants in the TOGETHER and *JEP* groups at post‐test. Because SEQ responses were anonymous, partners could not be linked within couples, and we were unable to account for dyadic interdependence.

## 4 Results

### 4.1 Descriptive Statistics and Comparison of Groups at Pre‐Test

Demographic characteristics of participants are presented in Table 1, along with test statistics comparing the two groups. Participants across groups significantly differed on several factors, including age, income, relationship status, level of education, parenting status, and proportion of same‐sex couples. *JEP* participants were older, had a lower monthly income, were more likely to be married, had less formal education, were more likely to be parents, and were less likely to be a same‐sex couple. Important to note is that most *JEP* participants were married (68%), had not finished high school (33%) or had a GED or high school diploma (26%), and reported no income or an income lower than $3000 (88%), which would be considered low‐income in the Washington, DC metropolitan area (U.S. Census Bureau 2024). The groups also differed in the number of workshop sessions they attended, with TOGETHER participants attending more than those in the *JEP* group (4.7 vs. 4.1 sessions, out of 6 total).

**TABLE 1:** Demographics by group.

	TOGETHER	JEP	Test statistic	Effect size
	Mean/Prop. (SD)	Mean/Prop. (SD)		
Number of sessions attended	4.72 (1.98)	4.13 (2.30)	4.58***	0.28
Age	36.31 (10.11)	38.85 (9.71)	−4.23***	0.26
Gender				
Female	51%	50%	0.26	0.02
Male	48%	50%		
Other	1%	0%		
Monthly income				
No earnings in the past 30 days	10%	29%	227.00***	0.46
$1–$499	6%	11%		
$500–$1000	8%	18%		
$1001–$2000	9%	16%		
$2001–$3000	16%	14%		
$3001–$4000	13%	6%		
$4001–$5000	16%	4%		
More than $5000	23%	2%		
Race/Ethnicity				
Native American/Alaska Native	< 1%	< 1%	1100***	0.99
Asian	8%	0%		
African American	61%	0%		
Native Hawaiian/Pacific Islander	1%	0%		
White	23%	1%		
Latino/a	0%	98%		
Others	8%	1%		
Relationship status				
Married	51%	68%	65.93***	0.25
Engaged	16%	18%		
Divorced	< 1%	< 1%		
Never married	< 1%	1%		
Steady basis relationship	29%	11%		
On and off relationship	3%	2%		
Education completed				
No degree	2%	33%	395.59***	0.61
GED	2%	4%		
High school	9%	22%		
Vocational/Tech	4%	14%		
Some college (No degree)	16%	10%		
Associate's	7%	2%		
Bachelor's	33%	13%		
Master's or higher	27%	3%		
Same‐sex couple	8%	4%	10.42***	0.09
Parents	57%	89%	139.91***	0.36

*Note:* *N* = 568 for TOGETHER and 532 for JEP. SD = Standard deviation. Test statistic for means is *t*, for proportions is χ^2^. Effect size is Cohen's d for *t*‐tests and Cramer's V for χ^2^.

*** *p* < 0.001.

Table 2 presents the pre‐treatment values of each of the eight treatment outcomes by treatment group. Participants in the *JEP* group had lower psychological distress, higher positive conflict management, lower negative conflict management, lower psychological aggression by and toward partner, higher relationship quality, and more difficulty paying bills. In sum, couples in the TOGETHER group had worse relationship functioning but better financial well‐being at pre‐treatment compared to couples in the *JEP* group.

**TABLE 2:** Comparison of pre‐test values in treatment outcomes by group.

		Pre‐test value	Cohen's d	Contrast	SE	z	*p*	95% CI
Psychological distress	TOGETHER	2.137	0.46	−0.230	0.050	−4.630	< 0.001	[−0.327, −0.133]
	JEP	1.907						
Positive conflict management	TOGETHER	3.020	0.13	0.278	0.044	6.370	< 0.001	[0.192, 0.363]
	JEP	3.297						
Negative conflict management	TOGETHER	2.548	1.17	−0.584	0.062	−9.440	< 0.001	[−0.706, −0.463]
	JEP	1.963						
Psychological aggression toward partner	TOGETHER	2.025	0.67	−0.334	0.056	−5.930	< 0.001	[−0.444, −0.224]
	JEP	1.691						
Psychological aggression by partner	TOGETHER	2.117	0.71	−0.358	0.066	−5.420	< 0.001	[−0.487, −0.228]
	JEP	1.760						
Time spent with partner	TOGETHER	3.333	0.18	0.088	0.050	1.760	0.078	[−0.010, 0.186]
	JEP	3.421						
Relationship quality	TOGETHER	3.179	0.64	0.319	0.047	6.800	< 0.001	[0.227, 0.411]
	JEP	3.497						
Difficulty paying bills	TOGETHER	1.949	0.65	0.323	0.063	5.080	< 0.001	[0.198, 0.447]
	JEP	2.272						

*Note:* *N* = 568 for TOGETHER and 532 for JEP.

Abbreviations: CI, confidence interval; SE, standard error.

### 4.2 Attrition

Overall, 65% of participants were retained at post‐test, though this differed by group, with 69% of participants retained in the TOGETHER group and 60% of participants retained in the *JEP* group. Across the full sample, participants who did not complete the post‐test differed from those who did on the number of workshop sessions attended, income, race/ethnicity, relationship status, education, same‐sex couple, and parenting status (see Table S2). Completers tended to attend more sessions, have higher income and education, be non‐Latine, be a same‐sex couple, and have fewer children. Except for reporting initial higher positive conflict management, completers did not differ from non‐completers in any other outcome variables at baseline.

### 4.3 Treatment Effects by Group

Results of each of the eight mixed models are reported in Table 3. The Group X Time interaction term is the key parameter which indicates whether there was a significant between‐group difference in change over time. Simple slopes that represent the treatment effect for each group are presented in Table 4. Compared to the TOGETHER group, the JEP group experienced an equivalent level of significant improvement in psychological distress, negative conflict management, psychological aggression toward partner, psychological aggression by partner, and time spent with partner. In addition, even though the JEP group experienced a significant increase in relationship quality, the magnitude of the change was smaller than in the TOGETHER group. Finally, unlike the TOGETHER group, the JEP group experienced no change in positive conflict management and did experience a significant decrease in difficulty paying bills.^3^

**TABLE 3:** Results of mixed models.

	Psychological distress		Positive conflict management		Negative conflict management		Psychological aggression toward partner		Psychological aggression by partner		Time spent with partner		Relationship quality		Difficulty paying bills	
	β	*p*	β	*p*	β	*p*	β	*p*	β	*p*	β	*p*	β	*p*	β	*p*
Treatment group	−0.23	< 0.001	0.06	0.053	−0.58	< 0.001	−0.33	< 0.001	−0.36	< 0.001	0.09	0.078	0.32	< 0.001	0.32	< 0.001
Time	−0.13	< 0.001	0.08	0.001	−0.24	< 0.001	−0.21	< 0.001	−0.21	< 0.001	0.10	0.001	0.15	< 0.001	−0.06	0.163
Group × Time	0.00	0.982	−0.10	0.006	0.09	0.088	0.07	0.233	0.02	0.703	−0.03	0.591	−0.07	0.045	−0.13	0.040
Constant	2.14	< 0.001	3.13	< 0.001	2.55	< 0.001	2.03	< 0.001	2.12	< 0.001	3.33	< 0.001	3.18	< 0.001	1.95	< 0.001

*Note:* *N* = 1100.

**TABLE 4:** Simple slopes of within‐person change in treatment outcomes by group.

		Effect size of between‐group comparison of slopes	Simple slope	SE	*z*	*p*	95% CI	Effect size of within‐group change
Psychological distress	TOGETHER	0.01	−0.132	0.031	−4.240	< 0.001	[−0.193, −0.071]	0.15
	JEP		−0.131	0.035	−3.770	< 0.001	[−0.199, −0.063]	0.14
Positive conflict management	TOGETHER	0.32	0.158	0.027	5.800	< 0.001	[0.105, 0.212]	0.09
	JEP		0.015	0.030	0.500	0.621	[−0.044, 0.074]	0.02
Negative conflict management	TOGETHER	0.12	−0.237	0.036	−6.630	< 0.001	[−0.307, −0.167]	0.27
	JEP		−0.145	0.040	−3.640	< 0.001	[−0.223, −0.067]	0.15
Psychological aggression toward partner	TOGETHER	0.10	−0.205	0.038	−5.360	< 0.001	[−0.281, −0.130]	0.23
	JEP		−0.137	0.043	−3.180	0.001	[−0.221, −0.052]	0.14
Psychological aggression by partner	TOGETHER	0.03	−0.212	0.041	−5.150	< 0.001	[−0.292, −0.131]	0.24
	JEP		−0.188	0.046	−4.070	< 0.001	[−0.278, −0.098]	0.20
Time spent with partner	TOGETHER	0.04	0.101	0.031	3.240	0.001	[0.040, 0.162]	0.11
	JEP		0.076	0.035	2.190	0.028	[0.008, 0.144]	0.08
Relationship quality	TOGETHER	0.13	0.148	0.025	6.000	< 0.001	[0.100, 0.197]	0.17
	JEP		0.074	0.028	2.670	0.008	[0.020, 0.144]	0.08
Difficulty paying bills	TOGETHER	0.16	−0.058	0.041	−1.400	0.163	[−0.139, 0.023]	0.07
	JEP		−0.185	0.046	−4.020	< 0.001	[−0.275, −0.094]	0.19

*Note:* *N* = 568 for TOGETHER and 532 for JEP. Effect sizes are Cohen's d. For the effect size of the between‐group comparison of slopes, it is calculated based on the estimated group difference in slopes divided by the square root of the pooled standard deviation of the slopes. For the effect size of the within‐group change from pre‐test to post‐test, it is calculated as the post‐test mean minus the pre‐test mean divided by the pre‐test standard deviation.

Abbreviations: CI, confidence interval; SE, standard error.

### 4.4 Satisfaction With the Program by Group

Regarding program helpfulness at post‐treatment, the *JEP* group rated the program significantly higher in overall helpfulness (Mean Diff = 0.43, SE = 0.08, *z* = 5.72, *p* < 0.001) and helpfulness for financial well‐being (Mean Diff = 0.43, SE = 0.08; *z* = 5.544, *p* < 0.001). *JEP* participants also gave significantly higher ratings, ranging from 4.82 to 4.88, on all aspects of the workshop, including the clarity, organization, and relevance of the information presented, the usefulness and appropriateness of activities, suitability of time and location, as well as overall satisfaction with the program (see Table S3). *JEP* participants also reported significantly higher ratings in terms of their acquired skills in communication (*t =* 3.25, df = 534, *p* < 0.01), stress management (*t =* 2.72, df = 534, *p* < 0.01), and financial management (*t =* 3.28, df = 533, *p* < 0.01). Additionally, *JEP* participants gave higher ratings to the relationship facilitators (*t =* 3.49, df = 534, *p* < 0.001) and financial facilitators (*t =* 3.51, df = 535, *p* < 0.001).

## 5 Discussion

In line with previous TOGETHER program evaluations (Azizi et al. 2025; Falconier 2015; Falconier, Kim, and Lachowicz 2023; Falconier, Foss, et al. 2023) and the *JEP* pilot test (Yumiseva et al. 2025), results from the present study provide preliminary evidence that participation in *JEP* was associated with improvements in individual well‐being, relationship functioning, and financial management among Spanish‐speaking Latine couples. Apart from positive conflict management, which showed no improvement, *JEP* participants demonstrated meaningful pre‐ to post‐intervention changes across most outcome domains, and such changes were either comparable to or significantly greater than those in the TOGETHER group. Furthermore, *JEP* participants reported significant decreases in difficulty paying bills, a change that was not observed in the TOGETHER participants.

These positive shifts align with *JEP*'s design, which focuses on strengthening couples' relational and financial well‐being within a stress‐management intervention framework. The observed reduction in psychological distress may reflect the program's emphasis on understanding stress and building coping skills at the individual and dyadic levels. Likewise, decreases in negative conflict management and psychological aggression, together with increases in time spent together and relationship quality, likely stem from opportunities to practice constructive communication, emotional regulation, and conjoint problem‐solving throughout the sessions. Improvements also extended to financial stress, suggesting that activities focused on budgeting and joint financial planning may have strengthened couples' collaboration and sense of control. In contrast, the absence of significant gains in positive conflict management suggests that for many couples, the most immediate benefits may come from reducing maladaptive patterns and increasing shared time together rather than expanding already moderate levels of positive communication.

These changes are particularly notable given that participants, on average, faced multiple vulnerabilities, including low income, limited formal education, and, in most cases, immigrant status, which often coincide with barriers to participation in CRE (Williamson et al. 2019) and contribute to their historical underrepresentation in CRE research (Tseng et al. 2021). *JEP*'s ability to engage these couples highlights the value of delivering CRE in participants' primary language and designing content that reflects their everyday realities. By explicitly addressing relational and contextual stressors, *JEP* illustrates how CRE can be adapted to the complex circumstances of diverse families. Although based on short‐term post‐program data, these results point to *JEP*'s promise as a culturally grounded approach for Spanish‐speaking Latine couples.

With these results, *JEP* represents one of the few, if not the only, CRE programs available entirely in Spanish that is socio‐culturally attuned to the experiences of Latine immigrant couples and supported by empirical evidence of effectiveness. Unlike direct translations of existing English‐language curricula, *JEP* was intentionally designed through a process of cultural adaptation that integrates culturally meaningful values and practices. For example, the program explicitly addresses financial realities common among immigrant families, such as remittances and multigenerational households. Equally important, *JEP* is grounded in Latine cultural values such as familismo, respeto, and personalismo, and attentive to contextual realities of migration, discrimination, and economic strain. Together, these features illustrate how fidelity to evidence‐based principles can coexist with deep cultural responsiveness. In doing so, *JEP* contributes to the broader CRE literature by demonstrating that culturally grounded interventions can produce meaningful change among couples for whom relationship education opportunities remain limited.

From a field perspective, these results contribute to ongoing discussions about how CRE can evolve to better serve diverse families (Markman et al. 2022). Culturally responsive programs like *JEP* can increase engagement and perceived relevance, key mechanisms that may ultimately enhance long‐term outcomes. The online delivery format also offers a scalable strategy to overcome barriers such as transportation, childcare, and scheduling constraints that often limit participation in traditional in‐person services (Williamson et al. 2019).

## 6 Limitations

Several limitations must be considered when interpreting these findings. First, the follow‐up assessments were conducted immediately following the final session and were completed primarily by participants who attended all sessions. Consequently, the results are reflective of short‐term changes among program completers and do not necessarily reflect sustained outcomes among all enrollees. Future research can more rigorously evaluate the *JEP* program's effectiveness with an RCT, randomly assigning Latine couples to either the *JEP* intervention or a control group and collecting long‐term follow‐up data. Additionally, reliance on funding agency‐mandated measures constrained the assessment of critical program domains, including financial stress and financial stress management. Moreover, the use of self‐report measures may have introduced social desirability bias. Future research could employ multimethod designs, such as observational methods, which were used in the *JEP* pilot study (Yumiseva et al. 2025), to measure behavioral changes that may not be fully reflected in self‐report data.

Another limitation concerns the lack of a reliable assessment of case management services, particularly regarding participants' use of the community services to which they were referred. Determining whether participants had followed up on the referrals provided was not always possible, which limited our ability to measure and disentangle the impact of the case management support from the effects of the program's educational components. Future research may benefit from the inclusion of a comparison group without case management to better isolate program effects. Finally, the overrepresentation of different‐sex couples restricts the generalizability of the results to same‐sex couples.

## 7 Implications and Conclusion

Overall, this study offers initial evidence that a socio‐culturally adapted, Spanish‐language CRE program can engage and benefit low‐income Latine couples. While results are encouraging, they should be interpreted as associations reflecting short‐term within‐person change, rather than as causal program effects. Nonetheless, *JEP's* success in producing measurable improvements in well‐being and relationship functioning demonstrates the promise of a culturally grounded CRE program. *JEP's* integrative model may inform future adaptations of CRE programs seeking to balance cultural relevance, accessibility, and fidelity to core evidence‐based principles. Future research should prioritize randomized controlled trials, long‐term follow‐ups, and multi‐site replication to confirm whether these short‐term gains translate into sustained improvements in relational and financial stability. As such, *JEP* represents an important step toward expanding the inclusivity and ecological validity of the CRE field, particularly for Latine couples.

## Funding

This study was partially funded (program implementation and data collection) by grant #90ZB000 from the Office of Family Assistance, Administration of Children and Families, U.S. Department of Health and Human Services.

## Conflicts of Interest

The authors declare no conflicts of interest.

## Supporting information


**Table S1:** TOGETHER program modules.
**Table S2:** Comparison of participants who did and did not complete post‐test.
**Table S3:** Session evaluation questions by group.
**Table S4:** Variance by workshop series/cohort, couple, and individual.

## Data Availability

The data that support the findings of this study are available on request from the corresponding author. The data are not publicly available due to privacy or ethical restrictions.

## Endnotes

^1^

*Familismo* is a cultural value central to Latine communities that emphasizes strong family bonds, loyalty, and interdependence among extended family members (Stein et al. 2014). It reflects a collective orientation in which family well‐being is prioritized over individual needs, influencing decision‐making, emotional support, and social obligations (Lugo Steidel and Contreras 2003).

^2^

*Personalismo* refers to Latines' tendency to value interpersonal relations and trust personal recommendations (Evans et al. 2007).

^3^
Given that there were significant between‐group differences in demographics (age, income, relationship status, level of education, parenting status, and proportion of same‐sex couples) and number of sessions attended, we added these variables as covariates in the main effects analyses to determine whether any of the differences in treatment effects were accounted for by these between‐group differences. Results all remained the same after adjusting for these covariates. Cohort had minimal impact on the outcomes as the well‐being variance explained by cohort ranged from 0% to 4% (Table S4).
