# Supplementary material for: Evaluation of the Individual, Relationship, and Financial Benefits of Juntos en Pareja for Spanish‐Speaking Latine Couples
Source: Fam Process. 2026 May 14;65:e70153. doi: 10.1111/famp.70153 (PMC13175748; doi:10.1111/famp.70153)
Supplement: Supplementary file 1 — Table S1: TOGETHER program modules. Table S2: Comparison of participants who did and did not complete post‐test. Table S3: Session evaluation questions by group. Table S4: Variance by workshop series/cohort, couple, and individual. [file FAMP-65-0-s001.docx]

**Supplemental Materials**

**Table S1**

*TOGETHER Program Modules*

| **Module** | **Name** |
| --- | --- |
| I | Introduction |
| 1 | Understanding Stress and Financial Stress |
| 2 | Improving Individual Stress and Financial Stress Management |
| 3 | Communicating Stress and Financial Stress to Your Partner |
| 4 | Improving Dyadic Stress and Financial Stress Management |
| 5 | Communicating Effectively and Learning to Talk about Money |
| 6 | Clarifying Financial Roles and Expectations |
| 7 | Improving Financial Management Skills |
| 8 | Credit and Risk Management |
| 9 | Improving Financial Problem-Solving Skills |
| R | Review |

| **Table S2**  *Comparison of Participants Who Did and Did Not Complete Post-test* | | | | | | |  |
| --- | --- | --- | --- | --- | --- | --- | --- |
|  | Did not complete | |  | Completed | |  |  |
|  | Mean/Prop. | *SD* |  | Mean/Prop. | *SD* | Test statistic | Effect size |
| Number of sessions attended | 1.75 | 1.24 |  | 5.94 | 0.28 | -85.73*** | 5.39 |
| Age | 37.17 | 9.66 |  | 37.73 | 10.18 | -0.87 | .05 |
| Gender |  |  |  |  |  | 0.07 | .01 |
| Female | 50% |  |  | 50% |  |  |  |
| Male | 50% |  |  | 49% |  |  |  |
| Other | 0% |  |  | 1% |  |  |  |
| Monthly Income |  |  |  |  |  | 19.57** | .14 |
| No earnings in the past 30 days | 24% |  |  | 16% |  |  |  |
| $1 - $499 | 7% |  |  | 9% |  |  |  |
| $500 - $1,000 | 15% |  |  | 11% |  |  |  |
| $1,001 - $2,000 | 11% |  |  | 13% |  |  |  |
| $2,001 - $3,000 | 16% |  |  | 15% |  |  |  |
| $3,001 - $4,000 | 7% |  |  | 11% |  |  |  |
| $4,001 - $5,000 | 9% |  |  | 11% |  |  |  |
| More than $5,000 | 12% |  |  | 14% |  |  |  |
| Race/Ethnicity |  |  |  |  |  | 14.88* | .12 |
| Native American/Alaska Native | 1% |  |  | <1% |  |  |  |
| Asian | 4% |  |  | 4% |  |  |  |
| African American | 28% |  |  | 33% |  |  |  |
| Native Hawaiian/Pacific Islander | 1% |  |  | 0% |  |  |  |
| White | 9% |  |  | 14% |  |  |  |
| Latino/a | 54% |  |  | 44% |  |  |  |
| Others | 4% |  |  | 4% |  |  |  |
| Relationship Status |  |  |  |  |  | 18.27** | .13 |
| Married | 56% |  |  | 61% |  |  |  |
| Engaged | 18% |  |  | 17% |  |  |  |
| Divorced | 0% |  |  | 0% |  |  |  |
| Never Married | 2% |  |  | 0% |  |  |  |
| Steady Basis Relationship | 21% |  |  | 20% |  |  |  |
| On and Off Relationship | 4% |  |  | 1% |  |  |  |
| Education completed |  |  |  |  |  | 29.17*** | .16 |
| No Degree | 23% |  |  | 13% |  |  |  |
| GED | 4% |  |  | 2% |  |  |  |
| High School | 17% |  |  | 14% |  |  |  |
|  | Did not complete | |  | Completed | |  |  |
|  | Mean/Prop. | *SD* |  | Mean/Prop. | *SD* | Test statistic | Effect size |
| Vocational/Tech | 8% |  |  | 9% |  |  |  |
| Some College (No degree) | 13% |  |  | 13% |  |  |  |
| Associate's | 4% |  |  | 5% |  |  |  |
| Bachelor's | 19% |  |  | 26% |  |  |  |
| Master's or higher | 13% |  |  | 17% |  |  |  |
| Same-Sex Couple | 4% |  |  | 7% |  | 4.83* | .07 |
| Parents | 81% |  |  | 68% |  | 20.07*** | .14 |
| Psychological Distress | 2.00 | 0.74 |  | 2.04 | 0.74 | -0.99 | -.06 |
| Positive Conflict Management | 3.09 | 0.53 |  | 3.20 | 0.44 | -3.41*** | -.22 |
| Negative Conflict Management | 2.30 | 0.91 |  | 2.26 | 0.86 | 0.74 | .05 |
| Psych. Aggression by Partner | 1.90 | 0.84 |  | 1.86 | 0.83 | 0.76 | .05 |
| Psych. Aggression toward Partner | 1.98 | 0.94 |  | 1.94 | 0.93 | 0.58 | .04 |
| Time Spent with Partner | 3.38 | 0.67 |  | 3.37 | 0.70 | 0.27 | .02 |
| Relationship Quality | 3.33 | 0.68 |  | 3.37 | 0.64 | -0.96 | -.06 |
| Difficulty Paying Bills | 2.10 | 0.96 |  | 2.10 | 0.92 | 0.01 | .00 |
| *Note.* N = 395 for did not complete and 705 for completed. *SD* = Standard deviation. Test statistic for means is t, for proportions is χ^2^. Effect size is Cohen’s d for *t* tests and Cramer’s V for χ^2^.  **p* < .05. ***p* < .01. ****p* < .001. | | | | | | | |

**Table S3**

*Session Evaluation Questions by Group*

| Item | TOGETHER | | JEP | | *df* | *t* | *p* |
| --- | --- | --- | --- | --- | --- | --- | --- |
|  | Mean | *SD* | Mean | *SD* |  |  |  |
| The information presented was clear. | 4.72 | 0.48 | 4.88 | 0.33 | 535 | 3.55 | <.001 |
| The information presented was well- organized. | 4.71 | 0.49 | 4.85 | 0.36 | 535 | 2.87 | 0.004 |
| The information presented was engaging. | 4.67 | 0.57 | 4.85 | 0.36 | 535 | 3.39 | <.001 |
| The information presented was relevant to my family situation. | 4.63 | 0.53 | 4.85 | 0.39 | 535 | 4.33 | <.001 |
| The activities/exercises for healthy relationships were appropriate. | 4.71 | 0.49 | 4.88 | 0.33 | 535 | 3.75 | <.001 |
| The activities/exercises for healthy relationships were useful. | 4.7 | 0.48 | 4.85 | 0.37 | 535 | 3.26 | 0.001 |
| The activities/exercises for financial education were appropriate. | 4.71 | 0.47 | 4.87 | 0.34 | 535 | 3.52 | <.001 |
| The activities/exercises for financial education were useful. | 4.7 | 0.51 | 4.87 | 0.36 | 535 | 3.48 | <.001 |
| The sessions were offered at a time that worked well for me. | 4.52 | 0.73 | 4.82 | 0.44 | 535 | 4.33 | <.001 |
| The sessions were offered at a location that worked well for me. | 4.71 | 0.58 | 4.87 | 0.34 | 535 | 2.91 | 0.004 |
| I would recommend the TOGETHER/JEP workshop to my friends or family. | 4.71 | 0.53 | 4.87 | 0.34 | 534 | 3.25 | 0.001 |
| As a result of my participation in the TOGETHER/JEP workshop, I have learned skills that I plan to implement into my communication. | 4.73 | 0.5 | 4.88 | 0.38 | 534 | 3.21 | 0.001 |
| As a result of my participation in the TOGETHER/JEP workshop, I have learned skills that I plan to implement into my stress management. | 4.72 | 0.5 | 4.85 | 0.37 | 534 | 2.72 | 0.007 |
| Item | TOGETHER | | JEP | | *df* | *t* | *p* |
|  | Mean | *SD* | Mean | *SD* |  |  |  |
| As a result of my participation in the TOGETHER/JEP workshop, I have learned skills that I plan to implement into my financial management. | 4.72 | 0.5 | 4.85 | 0.37 | 533 | 3.28 | 0.001 |
| As a result of my and my spouse’s/partner’s participation in the TOGETHER/JEP workshop, others can notice a positive change in us as a couple. | 4.72 | 0.5 | 4.85 | 0.37 | 534 | 4.5 | <.001 |
| Overall, I am satisfied with the TOGETHER/JEP Workshop. | 4.72 | 0.5 | 4.85 | 0.37 | 534 | 3.36 | <.001 |
| Across all sessions, how would you rate the performance of the couples facilitator? | 9.46 | 0.96 | 9.76 | 0.52 | 534 | 3.49 | <.001 |
| Across all sessions, how would you rate the performance of the financial facilitator? | 9.35 | 1.09 | 9.73 | 0.89 | 535 | 3.51 | <.001 |

**Table S4**

*Variance by Workshop Series /Cohort, Couple, and Individual*

|  | Psychological  Distress | | Positive Conflict Management | | Negative Conflict Management | | Psychological Aggression  Toward Partner | | Psychological Aggression  By Partner | | Relationship Quality | | Difficulty Paying Bills | |
| --- | --- | --- | --- | --- | --- | --- | --- | --- | --- | --- | --- | --- | --- | --- |
|  | Variance | ICC | Variance | ICC | Variance | ICC | Variance | ICC | Variance | ICC | Variance | ICC | Variance | ICC |
| Workshop Series/Cohort | 0 | 0% | 0.01 | 2% | 0.02 | 2% | 0.03 | 4% | 0.03 | 4% | 0.2 | 3% | 0 | 1% |
| Couple | 0.13 | 25% | 0.07 | 29% | 0.35 | 54% | 0.19 | 30% | 0.32 | 41% | 0.27 | 35% | 0.2 | 51% |
| Individual | 0.21 | 40% | 0.03 | 14% | 0.05 | 8% | 0.13 | 21% | 0.12 | 15% | 0.15 | 19% | 0.07 | 18% |
| Residual | 0.19 | 36% | 0.12 | 54% | 0.24 | 36% | 0.28 | 45% | 0.32 | 41% | 0.34 | 43% | 0.12 | 30% |
| TOTAL | 0.53 | 100% | 0.23 | 100% | 0.66 | 100% | 0.63 | 100% | 0.8 | 100% | 0.79 | 100% | 0.38 | 100% |
| *Note.* No convergence for Time Spent with Partner. | | | | | | | | | | | | | | |
